# From bench to bedside: targeting ferroptosis and mitochondrial damage in the treatment of diabetic cardiomyopathy

**DOI:** 10.3389/fendo.2025.1563362

**Published:** 2025-04-25

**Authors:** Bin Liu, Qing Jin, Yi Kang Sun, Zhi Ming Yang, Ping Meng, Xi Zhang, Qiu Chen, Pin Gan, Tao Zhao, Jia Ji He, Gui Ping He, Qiang Xue

**Affiliations:** ^1^ Department of Cardiology, The Fifth Affiliated Hospital of Kunming Medical University, Gejiu People’s Hospital, Gejiu, Yunnan, China; ^2^ Department of Cardiology, Yan’an Hospital Affiliated to Kunming Medical University, Key Laboratory of Cardiovascular Disease of Yunnan Province, Kun Min, Yunnan, China; ^3^ Yan’an Hospital Affiliated to Kunming Medical University, Key Laboratory of Cardiovascular Disease of Yunnan Province, Kun Min, Yunnan, China

**Keywords:** diabetic cardiomyopathy, ferroptosis, mitochondrial damage, molecular mechanisms, targeted therapy, combination therapy, clinical applications

## Abstract

Diabetic cardiomyopathy (DCM) is a common and fatal cardiac complication caused by diabetes, with its pathogenesis involving various forms of cell death and mitochondrial dysfunction, particularly ferroptosis and mitochondrial injury. Recent studies have indicated that ferroptosis and mitochondrial damage play crucial roles in the onset and progression of DCM, though their precise regulatory mechanisms remain unclear. Of particular interest is the interaction between ferroptosis and mitochondrial damage, as well as their synergistic effects, which are not fully understood. This review summarizes the roles of ferroptosis and mitochondrial injury in the progression of DCM and explores the molecular mechanisms involved, with an emphasis on the interplay between these two processes. Additionally, the article offers an overview of targeted drugs shown to be effective in cellular experiments, animal models, and clinical trials, analyzing their mechanisms of action and potential side effects. The goal is to provide insights for future drug development and clinical applications. Moreover, the review explores the challenges and prospects of multi-target combination therapies and personalized medicine interventions in clinical practice to offer strategic guidance for the comprehensive prevention and management of DCM.

## 1 Introduction

**Figure 1:**
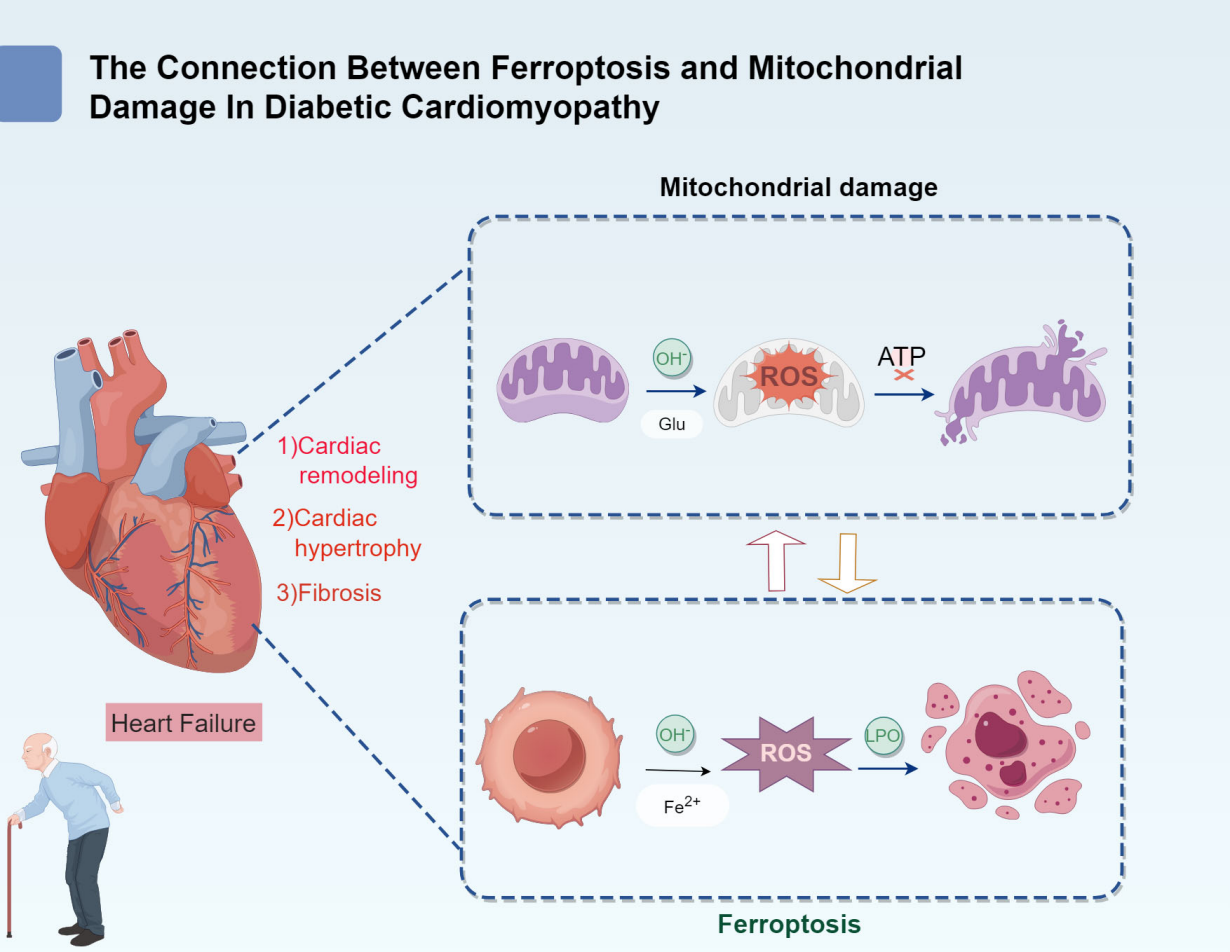
The connection between ferroptosis and mitochondrial damage in diabetic cardiomyopathy.

Diabetic cardiomyopathy (DCM) is a unique form of myocardial injury that occurs in patients with diabetes. It is not only closely linked to the systemic complications of diabetes but also involves specific pathological changes in myocardial metabolism and oxidative stress (1). Key factors contributing to DCM pathogenesis include disruptions in iron metabolism and mitochondrial dysfunction. The characteristic pathological features of DCM include lipid accumulation in myocardial cells, myocardial hypertrophy, myocardial fibrosis, mitochondrial damage, and myocardial ischemia (2). These alterations ultimately contribute to heart failure, which is a leading cause of mortality among patients with diabetes (**Figure 1**).

The increasing global prevalence of diabetes presents significant challenges to the prevention and treatment of DCM. According to the latest report by the International Diabetes Federation (IDF) (3, 4), the global prevalence of diabetes among adults aged 20–79 years had reached 14% by 2022, doubling the rate 30 years ago. The number of individuals with diabetes now exceeds 800 million, and this figure is projected to grow further in the coming decades. Early-stage cardiac damage in diabetic patients often goes unnoticed due to a lack of clear symptoms. Diagnosis typically occurs only when symptoms such as fatigue, shortness of breath, or edema appear after excluding other heart diseases (5). Due to its chronic and progressive nature, and the absence of specific early symptoms, early detection and intervention for DCM remain challenging. Research indicates that 20%–30% of diabetic patients may eventually develop DCM, and the risk of heart failure among these patients exceeds 70% (6, 7).

The pathogenesis of DCM is exceptionally complex, involving dysregulation of glucose and lipid metabolism, insulin resistance, oxidative stress, inflammatory responses, mitochondrial dysfunction, and ferroptosis (1, 8, 9). Among these, ferroptosis and mitochondrial damage have emerged as new areas of focus in recent years and are considered potential new therapeutic targets for DCM treatment. Ferroptosis is a form of programmed cell death induced by iron-dependent lipid peroxidation (10). Its core mechanisms include iron overload, lipid peroxidation, and disruption of the antioxidant defense system (11). Meanwhile, mitochondria, the cellular energy powerhouses, are directly involved in myocardial energy supply, and their dysfunction exacerbates myocardial impairment (12). The pathophysiological mechanisms of DCM are highly complex, involving factors such as metabolic disturbances in glucose and lipid homeostasis, insulin resistance, oxidative stress, inflammatory responses, mitochondrial dysfunction, and ferroptosis (1, 8, 9). Among these factors, ferroptosis and mitochondrial injury have emerged as key areas of focus and are considered potential therapeutic targets for DCM treatment (13).

Ferroptosis is a form of programmed cell death induced by iron-mediated lipid peroxidation (14). Its primary mechanisms involve iron overload, lipid peroxidation, imbalance in the antioxidant system, and the resulting cellular dysfunction (15). At the same time, mitochondria, which serve as the energy powerhouse of cells, are essential for maintaining cellular homeostasis. Mitochondrial dysfunction directly impairs myocardial energy supply, thereby exacerbating myocardial dysfunction (16). Ferroptosis has been observed in various organs, including the heart, liver, kidneys, brain, and retina of patients with diabetes (17–19). However, cardiac tissue is particularly vulnerable due to its high energy demands, unique metabolic characteristics (such as high mitochondrial density and continuous oxidative stress), and its critical role in physiological functions (20). This heightened vulnerability becomes more evident when there is damage to the diabetic heart, ventricular remodeling, and heart failure, where therapeutic strategies targeting ferroptosis could offer promising benefits. However, current studies on ferroptosis in diabetic cardiomyopathy are primarily restricted to limited clinical observations and animal experiments (21), underscoring the need for more comprehensive and systematic research.

This review will focus on exploring the role of ferroptosis and mitochondrial injury in cardiac cells exposed to chronic diabetic conditions, their impact on patients with diabetes, and the associated pathophysiological mechanisms. The main focus is to provide new strategies and recommendations for interventions targeting these mechanisms, as well as for personalized treatment approaches, combination therapies, and the development of innovative drug therapies.

## 2 Ferroptosis and its link to DCM

Ferroptosis is a form of programmed cell death driven by the excessive accumulation of iron ions within cells, triggering the Fenton reaction, activating oxidative stress, and disrupting lipid metabolism, ultimately leading to cellular demise (22, 23). The primary mechanisms underlying ferroptosis involve imbalances in iron metabolism and dysfunction of the antioxidant defense system. Glutathione (GSH), present in the cytoplasm of normal cells, is one of the most important low-molecular-weight antioxidants (24). It directly participates in scavenging reactive oxygen species (ROS) and reducing oxidative products, thus preventing oxidative stress-induced cellular damage (25). During ferroptosis, GPX4 binds to GSH to reduce lipid peroxides (such as PE-PUFA-OOH) to their corresponding alcohols, preventing lipid peroxide accumulation and protecting cellular membranes and structures from oxidative damage (20, 26).

Acyl-CoA synthetase long-chain family member 4 (ACSL4) is a key enzyme involved in the synthesis of polyunsaturated fatty acid phosphatidylethanolamine (PUFA-PE) (27). It catalyzes the acylation of polyunsaturated fatty acids (PUFAs) to form polyunsaturated fatty acid-CoA (PUFA-CoA), which is subsequently esterified by lysophosphatidylcholine acyltransferase 3 (LPCAT3) to form PUFA-CoA (28). The esterified PUFA-CoA then reacts with phosphatidylethanolamine (PE) to form PUFA-PEs. These PUFA-PEs play a fundamental role in maintaining cell membrane integrity; however, their oxidation leads to excessive lipid peroxide accumulation, ultimately triggering ferroptosis (29). In the context of DCM, chronic hyperglycemia disrupts fatty acid metabolism, leading to ACSL4 overexpression, increased PUFA-CoA production, and accelerated lipid peroxidation. This cascade not only elevates lipid peroxide levels but also intensifies iron accumulation in myocardial cells, exacerbating ferroptosis and contributing to myocardial cell death and fibrosis (30).

In patients with DCM, the GSH-dependent antioxidant mechanism is suppressed, whereas ACSL4 activation promotes ferroptosis in myocardial cells (31). The combined effects of oxidative stress and fatty acid metabolism disorder further exacerbate myocardial cell damage and promote ferroptosis (1, 8, 9). Unlike apoptosis, necrosis, or autophagy, ferroptosis exhibits distinct pathological features, including cell membrane rupture, mitochondrial dysfunction, and significant oxidative stress (32). Ferroptosis is particularly prominent in metabolic diseases, with its manifestation being more pronounced in DCM.

In the development of DCM, sustained hyperglycemia acts as a key driver of ferroptosis. Hyperglycemia disrupts myocardial fatty acid metabolism and reduces the capacity of myocardial cells to tolerate iron overload (33). Excessive accumulation of iron ions intensifies the production of reactive oxygen species (ROS) through the Fenton reaction, which, in turn, exacerbates lipid peroxidation. This process leads to a decrease in the activity of antioxidant enzymes such as glutathione peroxidase 4 (GPX4), superoxide dismutase 1 (SOD1), and NAD(P)H quinone oxidoreductase 1 (NQO1) (34). This decline in antioxidant enzyme activity further exacerbates ferroptosis (23, 33, 35), leading to uncontrolled lipid peroxidation, accelerating myocardial cell death, and triggering myocardial fibrosis and heart dysfunction (36). Among these, the inactivation of GPX4—a crucial inhibitor of ferroptosis—plays a significant role in the pathogenesis of myocardial injury in DCM.

Clinical and pathological studies have shown that patients with DCM exhibit significantly increased iron content in myocardial tissue, along with expansion of the cardiac chambers (37). In these patients, oxidative stress markers such as ACSL4, malondialdehyde (MDA), and NOX4 are generally elevated, whereas the expression of antioxidant enzymes like GPX4 and GSH is significantly reduced (36, 38). Furthermore, mitochondrial damage in myocardial cells of patients with DCM manifests as mitochondrial swelling, cristae disruption, and functional disturbances (32, 39). These pathological changes closely overlap with the characteristics of ferroptosis. These phenomena may be interrelated, further exacerbating myocardial damage and promoting the onset and progression of DCM.

### 2.1 Impact of glycemic disorder on ferroptosis in DCM and molecular regulatory mechanisms

In recent years, the dysregulation of glucose metabolism has emerged as a pivotal focus in research on DCM. Chronic fluctuations in blood glucose not only trigger oxidative stress but also promote the accumulation of advanced glycation end products (AGEs), exacerbating iron overload and lipid peroxidation (40). In DCM patients, impaired oxidative phosphorylation and the accumulation of AGEs in myocardial cells result in reduced ATP production. This energy deficiency makes cardiac cells more vulnerable to oxidative damage, particularly in high-glucose environments where iron overload is prevalent (41). Elevated blood glucose levels further increase the production of ROS, activating intracellular oxidative stress responses and further facilitating iron accumulation (42). Additionally, hyperglycemia promotes fatty acid synthesis pathways, enhancing the production of lipid peroxides, which accelerates ferroptosis in cardiac cells (33, 43).

Hyperglycemia promotes ferroptosis in myocardial cells through several interrelated mechanisms, which collectively contribute to the progression of DCM. These mechanisms include: (1) hyperglycemia induces an imbalance in the redox state of iron ions, promoting free radical generation and accelerating ferroptosis (44); (2) glucose dysregulation triggers iron ion accumulation, activating lipid peroxidation via the Fenton reaction, thereby promoting ferroptosis (23, 45); (3) chronic inflammation in diabetes exacerbates iron transport, storage, and utilization disorders, leading to the accumulation of free iron and inducing ferroptosis and myocardial fibrosis (46); (4) hyperglycemia disrupts mitochondrial energy supply, increasing ROS generation, further aggravating iron metabolic imbalance and eventually causing myocardial damage and ventricular remodeling (42, 47); (5) glucose dysregulation suppresses the expression of antioxidant genes via the Nrf2/ARE pathway, weakening the antioxidant defense and DNA repair capacity of the p53 pathway (48, 49); (6) insulin resistance, along with hyperglycemia, inhibits GPX4 activity, promotes AGEs formation, and disrupts fatty acid metabolism, ultimately accelerating the onset of ferroptosis (40, 50, 51).

### 2.2 Impact of iron homeostasis imbalance on ferroptosis in DCM and molecular regulatory mechanisms

Iron homeostasis refers is the regulation of iron intake, transport, storage, and excretion, which directly impacts cellular energy metabolism, antioxidant defense, and immune responses (46, 52). Hepcidin, a key hormone synthesized by the liver, regulates iron absorption and storage (52). Under normal physiological conditions, hepcidin reduces the expression of iron transport proteins like ferroportin, thereby limiting iron release and absorption to maintain iron homeostasis (53). Ferritin, a vital intracellular storage protein, sequesters excess iron to prevent its presence as free iron, thus reducing the risk of oxidative stress (54). Ferroportin facilitates iron transport by transferring intracellular iron into the bloodstream, thereby preventing iron accumulation in cells (55). When iron levels are high, ferroportin function is suppressed, leading to reduced iron release and its accumulation in cells. Additionally, transferrin receptor 1 (TfR1) mediates iron uptake by binding transferrin-bound iron and internalizing the complex via receptor-mediated endocytosis (56).

In DCM, blood glucose dysregulation triggers excessive oxidative stress and chronic inflammation, both of which are major contributors to ferroptosis (23). Hyperglycemia and insulin resistance promote oxidative stress and the release of inflammatory mediators, directly influencing the expression of hepcidin (13, 57). The upregulation of hepcidin inhibits the function of ferroportin, leading to intracellular iron accumulation (58), an increase in intracellular free iron levels, and subsequent lipid peroxidation and ferroptosis (59). Furthermore, chronic inflammation induces an increase in ferritin expression as a protective mechanism to store excess iron and reduce toxicity. However, excessive iron storage is insufficient to prevent iron overload, ultimately exacerbating ferroptosis.

Iron regulatory proteins (IRPs) are critical for maintaining iron homeostasis, as they control the expression of genes involved in iron absorption, storage, and export by interacting with iron response elements (IREs) (60). For instance, IRPs promote iron uptake by regulating TfR1 expression and increase iron storage by modulating ferritin synthesis (61). However, in diabetes, oxidative stress and chronic inflammation alter IRP activity, leading to iron metabolism dysregulation, iron overload, and increased ferroptosis (52). Thus, IRPs play a dual role in DCM: while they help maintain iron balance under normal conditions, their dysregulation in diabetes may contribute to cardiomyocyte ferroptosis and mitochondrial dysfunction (54, 55).

### 2.3 Role of oxidative stress in diabetic cardiomyopathy

In DCM, the disruption of iron homeostasis and persistent oxidative stress are key drivers of ferroptosis (52). These factors collectively contribute to lipid peroxidation, mitochondrial impairment, and ultimately, cardiomyocyte death (62). Several studies have shown that oxidative stress exacerbates ferroptosis through multiple signaling pathways, including System Xc-, Nrf2/KEAP1 pathway, and NOX4 (63).

**Figure 2:**
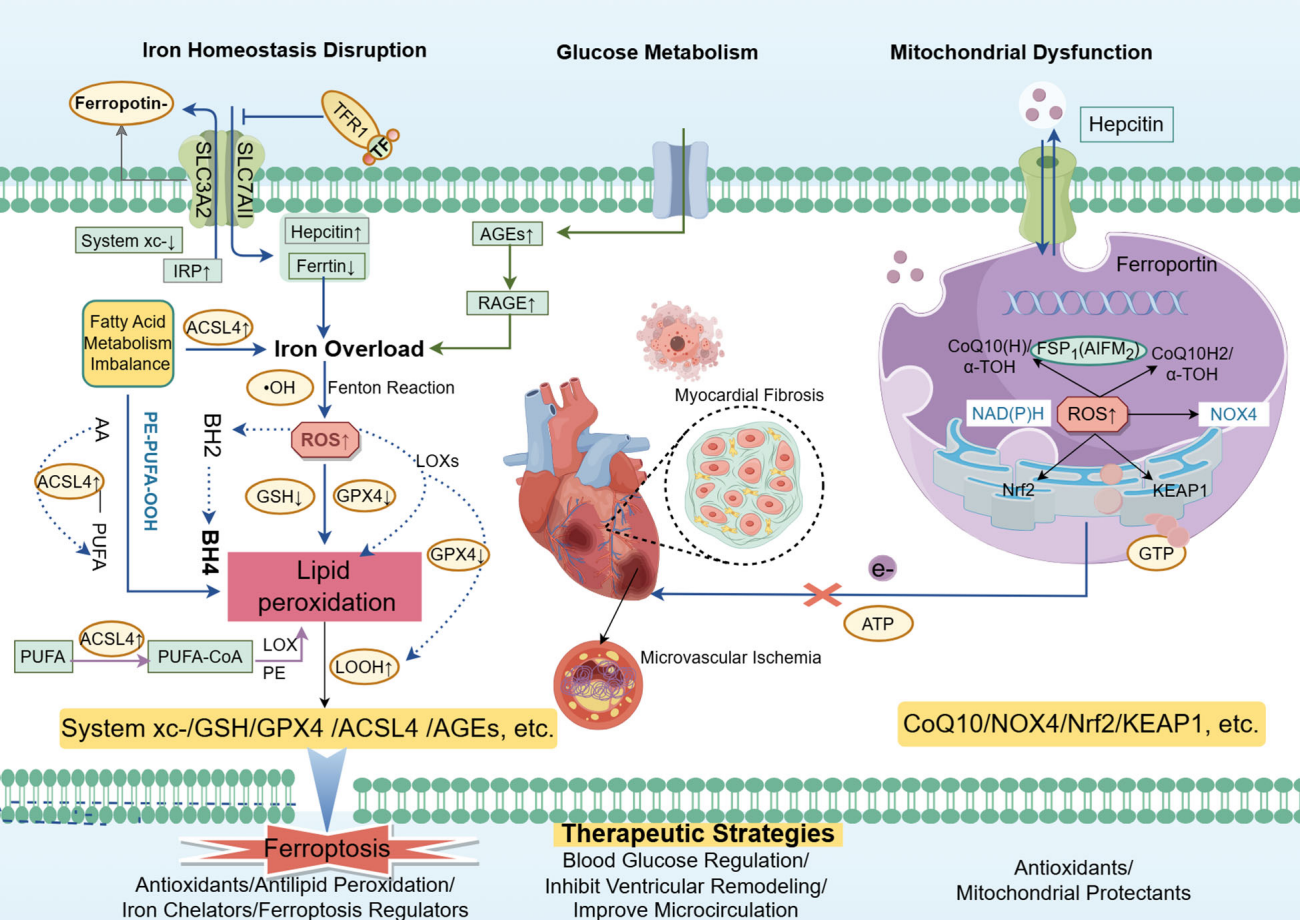
Ferroptosis and mitochondrial damage in diabetic cardiomyopathy (DCM) are driven by dysregulated glucose metabolism and insulin resistance, leading to iron overload and disturbed fatty acid metabolism in cardiomyocytes. This cascade results in excessive ROS generation and lipid peroxidation, activating pathways such as ACSL4 and hepcidin while inhibiting antioxidant systems like System Xc-, GSH, and GPX4. Iron overload and lipid peroxidation exacerbate mitochondrial dysfunction, impairing ATP synthesis and energy metabolism, ultimately contributing to cardiac fibrosis, heart failure, and the progression of DCM. Targeting ferroptosis and mitochondrial damage offers potential therapeutic strategies for managing DCM Courtesy of reference (70).

System Xc- is an essential amino acid antiporter composed of two subunits: SLC7A11 (solute carrier family 7 member 11) and SLC3A2 (solute carrier family 3 member 2) (64–66). This system imports cysteine, a precursor for GSH. GSH, a crucial antioxidant, effectively scavenges ROS to prevent lipid peroxidation and ferroptosis (31). A dysfunction in System Xc- results in reduced cysteine availability, which limits GSH synthesis, weakens antioxidant defenses, and increases susceptibility to lipid peroxidation, thereby accelerating ferroptosis (67). In DCM, System Xc- dysfunction further promotes ferroptosis, making it a critical molecular marker (68). The accumulation of advanced glycation end-products (AGEs) and oxidative stress caused by hyperglycemia further disrupts System Xc-, leading to iron deposition and exacerbated oxidative stress. This cascade directly promotes lipid peroxidation and ferroptosis (40, 67, 69) (**Figure 2**).

The nuclear factor erythroid-2-related factor 2 (Nrf2)/Kelch-like epichlorohydrin (ECH)-associated protein 1 (KEAP1) pathway plays a crucial role in DCM. Nrf2, a key transcription factor in oxidative stress response, is typically degraded by KEAP1 under normal conditions (71). However, under oxidative stress or pathological conditions like AGEs (72), Nrf2 is activated and translocated to the nucleus, initiating the expression of antioxidant genes such as GPX4, which are essential for reducing lipid peroxidation and delaying ferroptosis (73). Additionally, emerging research suggests that ferroptosis plays a significant role in diabetic retinopathy (DR) (74). Glucose dysregulation promotes upregulates long non-coding RNA ZFAS1, which enhances the expression of miR-7-5p. This cascade subsequently activates ACSL4, leading to increased lipid peroxidation and exacerbating oxidative damage and ferroptosis in retinal endothelial cells (75).

NOX4, a member of the NADPH oxidase family, interacts with Nrf2 and plays a significant role in oxidative stress (76). It catalyzes the production of superoxide and hydrogen peroxide, which increase lipid peroxidation, iron accumulation, and mitochondrial damage, thus driving ferroptosis (77). Under hyperglycemic conditions, NOX4 expression is significantly upregulated, further exacerbating ferroptosis (78). ROS generated by NOX4 also damage crucial components of the mitochondrial respiratory chain, further promoting mitochondrial dysfunction, which is a characteristic feature of DCM (79, 80).

#### 2.3.1 Summary

Disruption in iron homeostasis, coupled with oxidative stress and the involvement of critical signaling pathways such as System Xc-, Nrf2/KEAP1, and NOX4, plays a significant role in the induction of ferroptosis in DCM. Dysregulated iron metabolism further exacerbates myocardial damage, while the ZFAS1/miR-7-5p/ACSL4 axis promotes lipid peroxidation in DR, highlighting potential therapeutic targets.

## 3 Mitochondrial damage and its role in DCM

Disturbances in myocardial energy metabolism are a crucial factor in the development of DCM. Mitochondria, as the primary energy producers in cells, are central to cardiac energy production and function (47, 79). Mitochondrial dysfunction in DCM is often characterized by a loss of membrane potential, which inhibits ATP synthesis and triggers the release of cytochrome C and apoptosis-related factors, thereby promoting cardiomyocyte apoptosis or necrosis (81). Furthermore, insulin resistance (IR) and chronic hyperglycemia (CH) associated with diabetes exacerbate mitochondrial damage by increasing the production of reactive oxygen species (ROS) within mitochondria. This oxidative stress intensifies mitochondrial dysfunction, disrupts energy metabolism, and further promotes oxidative damage (82).

In a hyperglycemic environment, non-enzymatic glycation reactions are enhanced, leading to the formation of AGEs (40, 51, 83). AGEs not only react with biomolecules such as proteins and lipids but also exacerbate oxidative stress, impairing the energy metabolism of cardiomyocytes and ultimately leading to cardiac dysfunction (84). Oxidative stress can directly damage the mitochondrial structure, particularly the mitochondrial respiratory chain, severely limiting ATP synthesis (85, 86). This metabolic dysregulation reduces mitochondrial energy production efficiency, adversely affecting cardiac contraction and relaxation functions (87). Furthermore, these processes accelerate cardiomyocyte aging, compounding the risk of heart failure (50, 88). Coenzyme Q10 (CoQ10) plays a crucial role in maintaining cellular energy metabolism and ensuring normal electron transfer in the mitochondrial respiratory chain (89, 90). As a potent antioxidant, CoQ10 effectively scavenges excess ROS, thereby protecting cells from oxidative stress-induced damage (91). Moreover, CoQ10 shows considerable promise in inhibiting ferroptosis and reducing oxidative harm in cardiomyocytes. Ferroptosis Suppressor Protein 1 (FSP1) is a newly identified inhibitor of ferroptosis, functioning by catalyzing the reduction of membrane phospholipids to prevent the oxidation of PUFA-PE (92, 93). This action reduces lipid peroxidation, thereby slowing the progression of ferroptosis. The synergistic action between FSP1 and CoQ10 significantly suppresses oxidative stress, further inhibiting ferroptosis (94).

Overall, the metabolic disturbances associated with diabetes not only worsen mitochondrial dysfunction but also intensify oxidative damage to cardiomyocytes through ferroptosis. The interplay between these processes is a critical driver of DCM progression, ultimately leading to cardiac dysfunction (10). CoQ10, FSP1, and other mitochondrial protectants, with their antioxidant properties and ability to reduce lipid peroxidation, offer a promising therapeutic strategy to safeguard mitochondria, inhibit ferroptosis, and mitigate oxidative damage (91–93). This approach could provide new perspectives for the treatment of DCM.

### 3.1 Interaction between ferroptosis and mitochondrial damage in DCM

The interaction between ferroptosis and mitochondrial damage in DCM is a complex pathological process involving iron overload, mitochondrial energy metabolism disturbances, and oxidative stress. Studies have suggested that these factors promote myocardial cell damage and drive the pathological progression of DCM.

Iron overload is a key trigger of ferroptosis. In the diabetic environment, disruptions in glucose and lipid metabolism result in abnormal iron uptake and storage, particularly within cardiomyocytes, where iron accumulation is markedly increased (45, 79, 95). The imbalance in iron transporters and export proteins leads to intracellular iron buildup, with some of the excess iron entering the mitochondria. Once inside, iron participates in Fenton reactions, generating highly reactive free radicals, such as hydroxyl radicals. These radicals inflict severe cellular damage by disrupting membranes, proteins, and DNA, ultimately causing myocardial cell injury and death (96).

Iron accumulation not only directly damages cardiomyocytes but also exacerbates mitochondrial damage through the promotion of lipid peroxidation (13). Lipid peroxidation, part of the oxidative stress response, disrupts the lipid bilayer of cell membranes, impairing the integrity and function of mitochondrial membranes (97). These damages, in turn, promote the generation of additional free radicals, further exacerbating the oxidative damage to cardiomyocytes, thus creating a vicious cycle. Moreover, oxidative stress amplifies the ferroptosis process. Elevated levels of ROS damage membrane structures and regulate iron metabolism-related signaling pathways, including those involving hepcidin, ferritin, and iron transporters. This regulation increases iron release and facilitates further iron entry into cells (70). As ROS levels increase, mitochondrial function progressively deteriorates, leading to energy depletion and, ultimately, cardiomyocyte death.

In summary, the interaction between ferroptosis and mitochondrial damage in DCM forms a vicious cycle: from iron overload to oxidative damage and ultimately to cell death. This process not only aggravates myocardial cell injury but also accelerates the pathological progression of DCM (64). Therefore, targeting iron metabolism and mitochondrial protection may provide a novel strategy for the prevention and treatment of DCM.

### 3.2 Impact of ferroptosis and mitochondrial damage on cardiac structure and function in DCM

Ferroptosis and mitochondrial damage play a pivotal role in the pathogenesis of DCM, and their interaction exacerbates the metabolic crisis in the heart, significantly affecting cardiac structure and function (98). Research has shown that iron overload not only promotes the deposition of extracellular matrix (ECM) components such as collagen (99) but also activates signaling pathways like transforming growth factor-beta (TGF-β), triggering cardiac remodeling (100). Additionally, iron overload can activate inflammatory responses, initiating cell death pathways such as apoptosis and autophagy (32, 101, 102), further accelerating myocardial cell damage and death.

The oxidative stress induced by ferroptosis exacerbates mitochondrial damage, particularly by disrupting mitochondrial structure and function (85, 86, 103). This disruption severely impairs ATP synthesis in cardiomyocytes, leading to energy depletion. The subsequent energy metabolic disorder contributes to cardiac dysfunction, which may ultimately result in heart failure. Therefore, the interaction between ferroptosis and mitochondrial damage provides a mechanistic basis for the deterioration of cardiac function.

In patients with DCM, the interplay between ferroptosis and mitochondrial dysfunction significantly disrupts myocardial energy metabolism, exacerbates myocardial fibrosis, and accelerates the progression of heart failure (104). Studies conducted on animal models have demonstrated that treatment with iron chelators or antioxidants can effectively reduce iron buildup and ROS levels, leading to an improvement in cardiac function (105). These results highlight the importance of restoring iron balance and repairing mitochondrial function as key therapeutic strategies for enhancing cardiac health in DCM patients.

Given the critical roles of ferroptosis and mitochondrial dysfunction in the pathogenesis of DCM, developing targeted therapeutic strategies to restore iron balance and repair mitochondrial function may offer a promising approach for treating DCM. Such interventions could potentially improve cardiac function and provide new avenues for effective clinical management in the future.

## 4 Ferroptosis and mitochondrial injury interventions in DCM

### 4.1 Current treatment strategies for DCM and their limitations

Currently, treatment strategies for DCM primarily aim at controlling blood glucose levels and alleviating heart failure symptoms. Key therapeutic approaches include regulating blood glucose, managing blood lipids, improving insulin resistance, and preventing or treating cardiovascular and cerebrovascular complications induced by diabetes (106). Commonly used drugs in DCM and their mechanisms of action, as along with potential side effects are listed in **Table 1**. For example, metformin, a widely used antidiabetic drug, not only exerts its classic hypoglycemic effects but has also been shown to possess antioxidant and ferroptosis-inhibiting properties in recent studies (124). Metformin reduces the levels of lipid peroxidation products such as MDA and 4-hydroxy-2-nonenal (HNE), decreases intracellular ROS levels, and improves mitochondrial function, thereby inhibiting ferroptosis and alleviating the progression of DCM (125). Studies have demonstrated that metformin activates the AMP-activated protein kinase (AMPK) signaling pathway, improves iron homeostasis, enhances cellular antioxidant capacity, and helps mitigate cardiac damage associated with DCM (126, 127).

**Table 1:** Drugs for improving heart failure in DCM and alleviating cardiovascular and cerebrovascular complications.

Drug Class	Representative Drugs	Mechanism of Action	Adverse Effects	References
SGLT2 Inhibitors	Dapagliflozin, Empagliflozin, Canagliflozin	Inhibit sodium-glucose co-transporter 2 (SGLT2), improve insulin sensitivity, reduce myocardial fibrosis; reduce oxidative stress and enhance mitochondrial function, improving cardiac function in diabetic cardiomyopathy.	Hypotension, urinary tract infections, dehydration	(107–110)
GLP-1 Receptor Agonists	Semaglutide, Liraglutide	Activate GLP-1 receptors and improve glucose metabolism and cardiac function.	Nausea, vomiting, headache, pancreatitis	(111–113)
ACE/ARB Inhibitors	Valsartan, Ramipril, Lisinopril Valsartan Irbesartan	Inhibit vasoconstriction caused by angiotensin, reduce blood pressure, and reduce myocardial hypertrophy	Hyperkalemia, cough, dizziness, hypotension	(114)
Thiazolidinediones (TZDs)	Rosiglitazone, Pioglitazone	Activate PPAR-γ, improve insulin sensitivity, and reduce cardiac inflammation.	Weight gain, edema, fractures	(115, 116)
Biguanides	Metformin	Enhance insulin sensitivity, inhibit hepatic gluconeogenesis, and improve mitochondrial energy metabolism via the AMP-activated protein kinase (AMPK) pathway.	Gastrointestinal discomfort (e.g., nausea, diarrhea); rare cases of lactic acidosis and vitamin B12 deficiency	(117)
Renin-Angiotensin Inhibitors	Benazepril, Irbesartan	Inhibit the renin-angiotensin system, reduce blood pressure, and prevent fibrosis.	Hyperkalemia, dizziness, fatigue	(118, 119)
DPP-4 Inhibitors	Sitagliptin, Linagliptin	Inhibit DPP-4, increase GLP-1 levels, and reduce cardiac inflammation and fibrosis.	Upper respiratory tract infection, headache, dizziness	(112, 120)
Omega-3 Fatty Acids	Fish Oil, Eicosapentaenoic acid (EPA)	Reduce inflammation and improve cardiac lipid metabolism.	Fishy aftertaste, gastrointestinal discomfort, bleeding	(121)
Aspirin	Aspirin	Inhibit cyclooxygenase 1 and 2 (COX-1 and COX-2), reduce inflammation and thrombosis.	Gastrointestinal discomfort, bleeding	(122)
β-Blockers	Metoprolol, Bisoprolol	Block β-adrenergic receptors, reduce heart rate, and improve myocardial function.	Fatigue, bradycardia, hypotension	(123)

Sodium-glucose cotransporter 2 (SGLT-2) inhibitors, such as dapagliflozin and empagliflozin (107–110), work by inhibiting SGLT-2 in the kidneys, which decreases glucose reabsorption and lowers blood glucose levels. These drugs also exert diuretic effects, reduce cardiac inflammation, enhance myocardial energy metabolism, and alleviate oxidative stress, offering cardiac protection in DCM.

Glucagon-like peptide-1 receptor (GLP-1 receptor) agonists), such as liraglutide and semaglutide (111), mimic the action of endogenous GLP-1 to increase insulin secretion, suppress glucagon secretion, delay gastric emptying, and promote weight loss. In addition to improving blood glucose control, these drugs have been shown to improve cardiac structure, reduce myocardial hypertrophy, decrease cardiac fibrosis, and enhance heart function (112). They also help reduce vascular inflammation and oxidative stress.

Dipeptidyl peptidase-4 (DPP-4) inhibitors enhance insulin secretion and reduce glucose production by inhibiting DPP-4 enzyme activity (128). They also improve endothelial function and reduce cardiovascular burden (129). These medications represent a range of therapeutic options for preventing and treating DCM. However, current treatment strategies primarily focus on alleviating symptoms, such as controlling blood glucose and managing heart failure (130). A comprehensive treatment approach that targets the core pathological mechanisms of DCM, including myocardial energy metabolism dysregulation, mitochondrial dysfunction, and ferroptosis inhibition, has yet to be established.

While current treatments delay the progression of DCM to some extent, their effectiveness and safety remain limited. Notably, no specific medications have been identified that can significantly improve cardiac energy metabolism or repair mitochondrial function in DCM (131). Some studies suggest that certain drugs may have positive effects on cardiac metabolism and reduce myocardial injury, but their long-term efficacy and safety require further investigation (132). For example, the GLP-1 receptor agonist semaglutide has been shown to promote insulin secretion, improve cardiac metabolism, and reduce myocardial injury, thus delaying the progression of DCM (111). While preliminary results are promising, clinical data on the long-term effects and potential side effects of this drug remain insufficient, warranting further research and validation.

Moreover, new glucose level-lowering medications such as DPP-4 inhibitors (e.g., sitagliptin and vildagliptin) are widely used in diabetes treatment. *In vitro* and animal studies suggest that DPP-4 inhibitors can exert positive effects on DCM through mechanisms such as reducing cardiac inflammation and improving heart function (112, 120). However, the findings from relevant studies have been inconsistent. Some clinical trials report that DPP-4 inhibitors improve heart failure symptoms (133), while other studies suggest that their cardiac protective effects are limited (134). These discrepancies may be attributed to variations in study design, sample size, and trial standards. Therefore, future research should focus on prospective, multicenter, randomized controlled trials to further verify the efficacy and safety of DPP-4 inhibitors in DCM.

In conclusion, while current therapeutic approaches provide some relief in managing DCM, interventions targeting the disease’s core pathological mechanisms require further investigation. Future treatment strategies should place greater emphasis on addressing disorders in iron metabolism and mitochondrial damage to develop targeted therapies that offer more effective treatment options for patients with DCM.

### 4.2 Current Status of Targeted Ferroptosis and Mitochondrial Injury Treatment Strategies in DCM

As research into the pathogenesis of DCM progresses, ferroptosis and mitochondrial injury are increasingly recognized as key pathological contributors to the disease. Current targeted treatment strategies focus on iron chelators, antioxidants, mitochondrial protectants, and nanoparticle-based therapies (**Table 2**). These therapeutic approaches aim to reduce myocardial damage and prevent or delay the progression of DCM by targeting ferroptosis, repairing mitochondrial damage, and counteracting oxidative stress (85, 88, 103, 140). Ferroptosis inhibitors, particularly Ferrostatin-1, have been shown to effectively suppress ferroptosis under various pathological conditions (152, 176). Ferrostatin-1 exerts its action by inhibiting lipid peroxidation, particularly through its action on membrane lipids, thereby slowing the progression of ferroptosis (177). Additionally, Ferrostatin-1 exerts anti-inflammatory and antioxidant effects, further alleviating oxidative stress by inhibiting the Nrf2/ARE pathway and other antioxidant signaling pathways (178).

**Table 2:** Representative drugs for the improvement of ferroptosis and mitochondrial injury.

Category	Representative Drugs	Mechanism of Action	Drug Side Effects	References
Antioxidants and Ferroptosis Regulators	Vitamin C, Vitamin E	Antioxidants, neutralize free radicals, reduce oxidative stress, and protect the heart and mitochondrial membranes.	Headache, nausea, blurred vision; high doses may cause bleeding	(94)
	Nicotinamide	Modulates NAD+ levels, enhances cellular antioxidant capacity, and inhibits lipid peroxidation.	Gastrointestinal discomfort, nausea, vomiting, skin flushing; long-term use may cause gastrointestinal discomfort or liver damage	(135)
	N-Acetylcysteine (NAC)	Antioxidant, regulates iron metabolism to reduce free intracellular iron, protects cell membranes, improves mitochondrial injury, and reduces inflammation.	Gastrointestinal discomfort: nausea, vomiting, abdominal pain. Liver damage (long-term or high doses)	(136)
	Beta-Carotene	Scavenges free radicals, antioxidant; reduces lipid peroxidation, protects cell membrane structure.	Long-term high doses may lead to yellowing of the skin, increased risk of lung cancer	(137)
	Quercetin	Antioxidant, reduces oxidative stress; regulates iron metabolism, inhibits lipid peroxidation triggered by iron overload.	Long-term use may lead to gastrointestinal discomfort, allergic reactions	(138)
	Resveratrol	Activates SIRT1 pathway, reduces lipid peroxidation; regulates intracellular iron metabolism, prevents iron accumulation; modulates insulin sensitivity, reduces fat accumulation, and improves metabolic function.	Gastrointestinal symptoms like nausea and diarrhea; may interfere with anticoagulant activity, increasing bleeding risk; long-term high doses may cause indigestion and liver dysfunction	(139)
	Resveratrol Nanoparticles	Significantly improves mitochondrial function, antioxidant activity, and miR-20b-5p signaling pathway to improve symptoms.	Undetermined, possibly gastrointestinal discomfort like nausea, vomiting, or diarrhea; long-term safety remains unclear	(140, 141)
	Hesperidin	Antioxidant, anti-inflammatory; improves iron metabolism and reduces lipid peroxidation.	High doses may cause headache and nausea	(142)
Iron Chelators	Deferoxamine (DFO)	Chelates iron ions, reduces free iron, alleviates oxidative stress	Hearing loss, skin fibrosis, allergic reactions; long-term use may cause kidney damage	(122, 123, 143)
	Deferasirox (DFX)	Chelates iron ions, reduces iron accumulation, and alleviates ferroptosis	Gastrointestinal discomfort, liver function impairment, kidney dysfunction, rash	(144)
	Deferiprone (DFP)	Chelates iron ions, reduces iron accumulation, and alleviates oxidative stress.	Leukopenia, collagen deposition, gastrointestinal discomfort	(145)
	Hepcidin Mimetics	Mimics hepcidin to regulate iron metabolism, reduces iron accumulation, and inhibits oxidative stress.	Unknown	(146)
	Dexrazoxane (DEX)	Chelates excess iron in the body, reduces iron accumulation, inhibits lipid peroxidation, and alleviates heart damage caused by doxorubicin	Possible liver function impairment, gastrointestinal discomfort	(147)
	CN128	Binds free iron to form stable complexes that are excreted, preventing iron oxidation, free radical generation, and lipid peroxidation.	May cause gastrointestinal discomfort, constipation, and liver function impairment	(148)
	Ciclopirox(CPX)	Chelates free iron ions and inhibits iron-dependent oxidative stress responses	Rare gastrointestinal discomfort, nausea, and headaches.	(149, 150)
Ferroptosis Lipid Metabolism Regulators	Ferrostatin-1 (Fer-1)	Inhibits lipid peroxidases (such as ACSL4) and antioxidant protection to inhibit ferroptosis	May interfere with normal cell metabolism and cause cell death, suppress the immune system	(151–153)
	Liproxstatin-1	Inhibits lipid peroxidation and reduces ferroptosis	May affect cell metabolism and lipid disorders; excessive oxidative stress may damage cells and affect immune function	(154)
	Glutathione Peroxidase 4 Inhibitors (GPX4 Inhibitors)	Antioxidant enzyme activity, reduces lipid peroxidation, and prevents ferroptosis	Some cytotoxicity and immunosuppressive effects; excessive use may lead to liver, kidney, and nervous system damage	(155)
	Inhibitors of ACSL4	Inhibits ACSL4, reduces the synthesis and oxidation of polyunsaturated fatty acids, and slows or inhibits ferroptosis	Disturbs fatty acid metabolism, damages cell membrane structure, impairs mitochondrial function	(156)
	Tannic Acid	Inhibits lipid peroxidation, reduces iron overload; enhances antioxidant enzyme GPX4 activity	High doses may cause liver toxicity, inhibit iron absorption, leading to iron deficiency anemia	(157, 158)
	Allicin	Inhibits lipid peroxidation, reduces ferroptosis; activates antioxidant enzyme systems	High doses may cause gastrointestinal discomfort and allergic reactions	(159, 160)
	Azadirachtin	Inhibits oxidative stress, protects the cardiovascular system; anti-inflammatory, reduces lipid peroxidation.	Long-term use may cause liver toxicity and neurotoxicity	(161)
Ferroptosis Antioxidant and Mitochondrial Protectors	MitoQ	Mitochondria-targeted antioxidant, scavenges ROS, and reduces mitochondrial damage.	Generally well tolerated, but some individuals may experience mild gastrointestinal discomfort or skin irritation	(162, 163)
	Coenzyme Q10	Enhances mitochondrial energy production, reduces oxidative stress	May lead to overstimulation, causing other metabolic disorders	(164)
	Taurine	Antioxidant and osmotic regulator, protects cells from oxidative damage, and promotes mitochondrial function.	Mild side effects including headache and dizziness; may interact with other drugs	(165)
	Resveratrol	Antioxidant and anti-inflammatory properties, regulates mitochondrial function, and reduces ROS generation to protect from oxidative damage	Gastrointestinal discomfort, headache; high doses may cause liver enzyme abnormalities	(166)
	L-Carnitine	Promotes long-chain fatty acids into mitochondria for β-oxidation, thus enhancing energy production and reducing oxidative stress	Gastrointestinal discomfort (such as nausea, diarrhea), long-term use may lead to cardiovascular side effects	(167)
α2-Adrenergic Receptor Agonists	Dexmedetomidine (DEX)	Activates Nrf2/GPX4 pathway, inhibits ferroptosis, protects myocardium	May cause hypotension and sedative effects	(168, 169)
ATP-sensitive Potassium Channel Openers	Nicorandil	Activates ATP-sensitive potassium channels (KATP) in myocardial cells, reduces oxidative stress, improves mitochondrial function, reduces myocardial ferroptosis, and mitigates damage in diabetic cardiomyopathy	May cause headache, facial flushing, and hypotension	(170)
Hydrogen Sulfide Donor Drugs	Hydrogen Sulfide (H_2_S)	Increases glutathione and GPX4 activity and reduces lipid peroxidation and ROS production	Not well detailed; may cause respiratory discomfort and hypotension	(171)
	Sodium Hydrosulfide(NaHS)	Regulates calcium homeostasis in myocardial cells, improves contraction and relaxation function in DCM; improves mitochondrial apoptosis, modulates inflammatory response	May cause gastrointestinal discomfort, nausea, or vomiting; excessive use may lead to hypotension or arrhythmias	(172, 173)
Others	6-Gingerol Nanoparticles	Activates Nrf2/HO-1 pathway to alleviate ferroptosis and inflammation; improves heart function in DCM	Unknown	(174)
	β-Lapachone	Activates NQO1, enhances antioxidant capacity and mitochondrial function, and modulates ferroptosis	Unknown	(175)

Iron chelators are also essential in managing DCM. Deferoxamine, a widely used iron chelator, has proven effective in treating iron overload-related disorders but carries significant side effects, particularly affecting renal function and the immune system (179, 180). Deferiprone, another iron chelator, offers better oral bioavailability but still poses risks, including kidney toxicity. On the other hand, the novel oral iron chelator CN128 has shown superior iron chelation and antioxidant properties in clinical trials (148). It also has a longer half-life, fewer side effects, and is able to effectively reduce body iron overload without causing kidney damage (181). Additionally, other iron chelators, such as Ciclopirox (CPX) (149), have demonstrated the potential to inhibit iron-dependent oxidative stress, reduce lipid peroxidation, and improve myocardial cell health, offering enhanced myocardial protection in DCM. Furthermore, resveratrol nanoparticles have shown promising effects in protecting the heart by mitigating mitochondrial damage and inhibiting lipid peroxidation reactions (89).

Additionally, resveratrol nanoparticles have demonstrated protective effects on the heart by alleviating mitochondrial damage and inhibiting lipid peroxidation (182). These therapies function through multiple mechanisms, including modulating intracellular redox balance and improving mitochondrial function, offering new possibilities for the treatment of DCM.

Although these therapeutic strategies have shown promising results in laboratory settings, most studies are still limited to cell experiments, animal models, and a few clinical case reports. Comprehensive experimental data on drug mechanisms of action, pharmacodynamics, toxicology, and pharmacokinetics remain scarce (183). Furthermore, there is a lack of large-scale, multicenter, prospective preclinical studies and randomized clinical trials, meaning the effectiveness and safety of these treatment strategies have not been fully validated in clinical practice (151).

Therefore, while ferroptosis and mitochondrial injury-targeted therapies show great promise in the treatment of DCM, further in-depth exploration of the drugs’ mechanisms of action, efficacy, and safety is crucial to advance the clinical translation of these therapeutic approaches.

## 5 Prospects for basic research and clinical applications of therapeutic strategies for DCM

**Figure 3:**
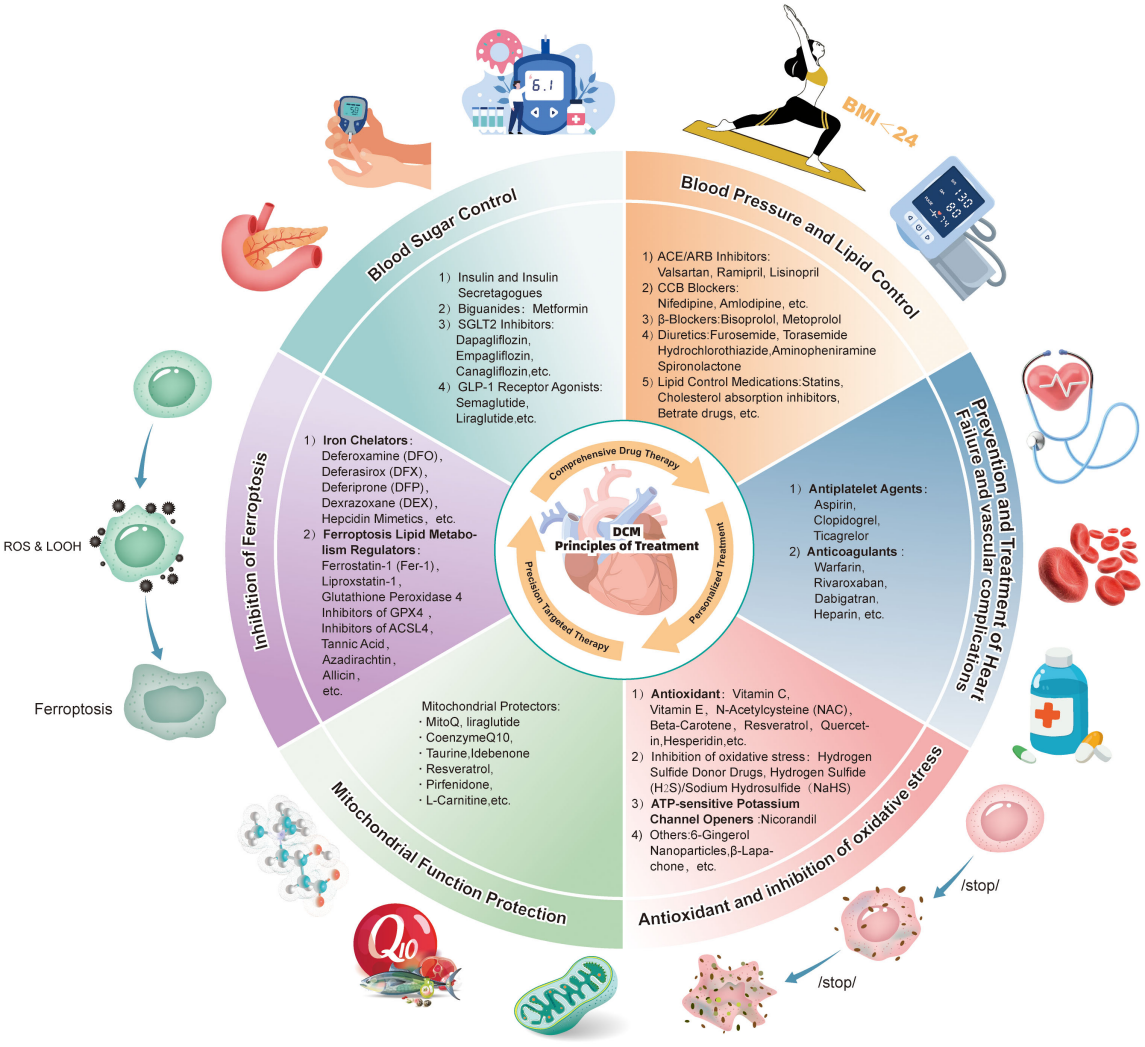
Comprehensive prevention and treatment strategies for diabetic cardiomyopathy: A schematic diagram of drug regimens based on combined pharmacological therapy, individualized treatment, and precision-targeted therapy targeting ferroptosis and mitochondrial damage.

The pathogenesis of DCM is multifaceted, involving ferroptosis, oxidative stress, mitochondrial injury, and other factors. Advancements in understanding these pathological processes are paving the way for innovative therapeutic strategies, particularly those centered on multi-target combination therapies and personalized medicine. Future therapeutic approaches may combine ferroptosis-targeted agents with mitochondrial protectants to achieve synergistic effects in alleviating myocardial injury and improving cardiac function (**Figure 3**).

### 5.1 Potential of combination therapy

The intricate pathological mechanisms of DCM present significant challenges for single-treatment approaches to address all aspects of the disease comprehensively. The interaction between ferroptosis and mitochondrial injury creates a synergistic effect, which exacerbates cardiac dysfunction during DCM progression (184). Consequently, combination therapies that target both ferroptosis and mitochondrial dysfunction have emerged as a promising direction for future clinical treatment. For example, the combined use of iron chelators and antioxidants has shown potential to effectively reduce iron overload while inhibiting oxidative stress, thereby offering enhanced protection for myocardial cells (136, 137, 143, 145). Similarly, combining GPX4 agonists with sodium-calcium exchanger inhibitors could suppress lipid peroxidation and safeguard mitochondrial integrity, thereby delaying myocardial cell death (185, 186). These combination strategies demonstrate strong synergistic effects, laying a robust theoretical foundation for the development of personalized treatment strategies. By tailoring treatment regimens based on an individual’s pathological state, these approaches could improve therapeutic efficacy while minimizing side effects.

With advancements in precision medicine, identifying specific pathological mechanisms in patients will be instrumental in guiding the development of targeted therapies. This approach would ensure that interventions are both effective and safe, addressing the variability in drug responses among patients (187). Ultimately, combination therapies grounded in multi-target and personalized strategies are poised to play a transformative role in the clinical management of DCM, offering hope for more effective and patient-specific treatments in the future.

### 5.2 Challenges and progress in clinical research

Although novel therapies such as ferroptosis inhibitors and mitochondrial protectants have shown promising efficacy in preclinical studies (164, 166, 188), their clinical translation still faces numerous challenges. Firstly, long-term drug efficacy evaluation and drug resistance are major difficulties in clinical trial design (189). The slow progression of DCM necessitates extended trial durations, thereby escalating costs and adding complexity to the research process (190). Furthermore, variability among patients, including genetic factors and individual pathological conditions, can profoundly influence drug responses, presenting a considerable hurdle in developing personalized treatment strategies (191).

Moreover, the combination of iron chelators and mitochondrial protectants has shown promising results in some studies (192), but several challenges remain, including issues related to drug formulation, safety, and potential interactions between medications. For instance, iron chelators such as deferoxamine and deferasirox effectively reduce iron overload and improve iron-related damage. However, prolonged or high-dose usage can lead to adverse effects such as liver and kidney toxicity or allergic reactions (122, 123, 179, 180). Therefore, the clinical use of these agents requires careful management, with dosages and treatment durations tailored to the patient’s specific condition.

Additionally, while drugs like SGLT2 inhibitors and GLP-1 receptor agonists have demonstrated benefits in improving cardiac function and glucose metabolism in patients with DCM (107, 108, 111, 113), their side effects present significant limitations in clinical practice. For example, SGLT2 inhibitors may cause hypoglycemia and urinary tract infections, whereas GLP-1 receptor agonists may trigger nausea and pancreatitis (193). Thiazolidinediones (e.g., rosiglitazone and pioglitazone) enhance insulin sensitivity and reduce cardiac inflammation but are linked to side effects such as weight gain, edema, and bone fractures with long-term use (115, 116, 194). These issues are particularly concerning for elderly patients and those with osteoporosis, who require careful evaluation of the risks and benefits of such medications (195). These concerns are particularly relevant for elderly patients and those with osteoporosis, necessitating a thorough assessment of risks and benefits.

### 5.3 Future research directions

As the pathogenesis of DCM continues to be explored, future research is likely to focus on several key areas:

#### 5.3.1 Development of precision-targeted drugs

Current pharmacological treatments for DCM and its complications largely rely on non-specific approaches, focusing on symptom management rather than addressing the underlying pathological mechanisms. Moving forward, research will prioritize the development of drugs that specifically target key processes such as ferroptosis and oxidative stress (14, 196). For example, therapeutics that modulate iron metabolism by targeting proteins like hepcidin or ferritin are anticipated to gain prominence in future studies. Additionally, compounds aimed at restoring mitochondrial function, particularly those capable of enhancing ATP synthesis or mitigating the generation of ROS, represent promising clinical advancements (197). These targeted therapeutic strategies have the potential to not only improve treatment outcomes but also pave the way for personalized therapeutic approaches tailored to the unique pathological conditions of individual patients.

#### 5.3.2 Development of novel drugs

Mitochondrial dysfunction is a central pathological feature of DCM, and developing drugs that restore mitochondrial function holds significant therapeutic potential (198). Agents that enhance ATP synthesis or reduce the generation of ROS are among the promising candidates for addressing this dysfunction (199). In addition to mitochondrial protectants, anti-inflammatory drugs may also offer new treatment avenues for DCM. Specifically, drugs that target inflammation pathways unique to the heart, such as those modulating inflammatory responses associated with myocardial fibrosis or cardiomyocyte (200).

#### 5.3.3 Drug combination strategies

Many monotherapeutic strategies have failed to yield the desired outcomes when used alone, prompting increasing interest in combination therapies. Current research indicates that combining SGLT2 inhibitors with GLP-1 receptor agonists has shown synergistic effects (67–69, 76). Future studies are likely to explore additional combination therapies, particularly those involving drugs with distinct mechanisms of action, such as pairing iron chelators with antioxidants or mitochondrial protectants (64). These combinations could improve therapeutic efficacy while minimizing side effects (201, 202). By targeting multiple pathological processes simultaneously, drug combinations offer a more comprehensive approach to treating DCM.

#### 5.3.4 Gene and stem cell therapies

With the ongoing progress in gene editing and stem cell research, gene therapy and cell-based treatments hold the potential to deliver groundbreaking therapeutic options for DCM (203). Gene editing technologies that repair mitochondrial function or stem cell therapies aimed at repairing myocardial damage could offer innovative solutions in the future. Gene therapy can address the molecular root causes of DCM, while stem cell therapy shows promise in promoting myocardial regeneration and functional recovery. These approaches could provide new avenues for the treatment of DCM, offering hope for improved outcomes for patients (204).

#### 5.3.5 Personalized medicine and precision healthcare

Future research should focus on personalized treatment strategies based on genomics, metabolomics, and other technologies. The pathological mechanisms may vary from patient to patient, and thus, precision medicine will play a key role in developing individualized therapeutic approaches. This will ensure that drug treatments are tailored to a patient’s specific pathological state, thereby improving efficacy and reducing side effects (205). Personalized medicine will not only enhance treatment outcomes but also reduce unwanted side effects, optimizing the overall treatment outcome for patients.

### 5.4 Clinical translation and challenges

Despite significant advancements in basic research for DCM treatment, translating these findings into clinical applications remains fraught with challenges. Firstly, although many drugs show promising results in animal models, there is a lack of sufficient large-scale clinical trial data to support their efficacy and safety across diverse patient populations (206). Thus, future research will require multi-center, large-scale randomized controlled trials to validate the feasibility and effectiveness of these drugs in clinical settings. Moreover, the combination of novel drugs with existing treatment modalities (e.g., SGLT2 inhibitors with ferroptosis inhibitors, antioxidants like selenocysteine with Ferrostatin-1 or Liproxstatin-1, and GLP-1 receptor agonists with ferroptosis inhibitors) (207–209) will be an important area of future research. Investigating the potential synergistic effects and interactions between these drugs will help optimize treatment regimens and improve overall efficacy (205, 210). Combination therapies may not only enhance therapeutic outcomes but also provide more treatment options to meet individual patient needs.

## 6 Conclusion

In conclusion, treating diabetic cardiomyopathy remains a complex challenge. While current medications can alleviate symptoms and slow the progression of the disease, their efficacy and safety still require further enhancement. Future research should prioritize precision medicine, aiming to minimize drug side effects while optimizing the effectiveness of multi-target combination therapies. With technological advances, the development of drugs targeting ferroptosis and mitochondrial dysfunction, personalized treatment strategies, and the integration of stem cell and gene therapies could pave the way for significant breakthroughs in the clinical management of diabetic cardiomyopathy.

## Glossary

DCM: Diabetic cardiomyopathy
IDF: International Diabetes Federation
ROS: Reactive oxygen species
GSH: Glutathione
GPX4: Glutathione peroxidase 4
AGEs: Advanced glycation end products
Nrf2: Nuclear factor erythroid 2-related factor 2
ARE: Antioxidant response element
Tf: Transferrin
Ft: Ferritin
Fpn: Ferroportin
IRPs: Iron regulatory proteins
DMT1: Divalent Metal Transporter 1
System Xc-: Cystine/Glutamate Antiporter System Xc-
TGF-β: Transforming growth factor-beta
IR: Insulin resistance
CH: Chronic hyperglycemia
ATP: Adenosine triphosphate
ECM: Extracellular matrix
SGLT-2: Sodium-glucose cotransporter 2
GLP-1: Glucagon-like peptide-1
DPP-4: Dipeptidyl peptidase-4
ACE: Angiotensin-converting enzyme
ARB: Angiotensin receptor blockers
TZDs: Thiazolidinediones
AMPK: Adenosine monophosphate-activated protein kinase
RAS: Renin-Angiotensin Inhibitors
EPA: Eicosapentaenoic acid
COX-1 and COX-2: Cyclooxygenase 1 and 2
NAC: N-Acetylcysteine
DFO: Deferoxamine
DFX: Deferasirox
DFP: Deferiprone
DEX: Dexrazoxane
Fer-1: Ferrostatin-1
GPX4 Inhibitors: Glutathione Peroxidase 4 Inhibitors
DEX: Dexmedetomidine
H₂S: Hydrogen Sulfide
NaHS: Sodium Hydrosulfide
NAD(P)H: Nicotinamide Adenine Dinucleotide (Phosphate)
NQO1: Quinone Oxidoreductase 1
GPT: Glutamate Pyruvate Transaminase
NOX4: NADPH Oxidase 4
REAP1: Receptor Interacting Protein 1
ACSL4: Acyl-CoA Synthetase Long Chain Family Member 4
SLC3A2: Solute Carrier Family 3 Member 2
SLC7A11: Solute Carrier Family 7 Member 11
PUFA: Polyunsaturated Fatty Acids
PE-PUFA-OOH: Phosphatidylethanolamine Polyunsaturated Fatty Acids
SOD1: Superoxide Dismutase 1
Nrf2: Nuclear factor erythroid-2-related factor 2
KEAP1: Kelch-like epichlorohydrin (ECH)-associated protein 1
